# Nesting biology and phylogenetic relationships of the parasitoid-hunting wasp *Lindenius pygmaeus armatus* (Vander Linden, 1829) (Hymenoptera: Crabronidae)

**DOI:** 10.1038/s41598-023-36019-1

**Published:** 2023-06-14

**Authors:** Piotr Olszewski, Eduardas Budrys, Svetlana Orlovskytė, Bogdan Wiśniowski, Toshko Ljubomirov

**Affiliations:** 1 Faculty of Biology and Environmental Protection, Natural History Museum, University of Łódź, Kilińskiego 101, 90-011 Łódź, Poland; 2 Institute of Ecology, Nature Research Centre, Akademijos 2, 08412 Vilnius, Lithuania; 3 Institute of Agricultural Sciences, Land Management and Environmental Protection, University of Rzeszów, 1 Ćwiklińskiej Str, 35-601 Rzeszów, Poland; 4 Bulgarian Academy of Sciences, Institute of Biodiversity and Ecosystem Research, Tzar Osvoboditel Boulevard 1, 1000 Sofia, Bulgaria

**Keywords:** Zoology, Animal behaviour, Entomology

## Abstract

Nests of *Lindenius pygmaeus armatus* were examined in northern Poland in Kowalewo Pomorskie and Sierakowo. Adults were encountered from late May to late July. The nests were built in sandy areas and wasteland. Seven nests were observed, of which two were dug up and their structure was examined. The channel was approximately 2.5 mm in diameter and 8–10 cm in the length. The material removed during digging was placed near the nest entrance. The main burrow led to 3–5 cells. The cocoons were approximately 5–7 mm long and 2.5–3.5 mm wide. Females of *L. p. armatus* provided their nest cells with chalcid wasps averaging 14 prey items per cell. Parasitoids *Myrmosa atra* and kleptoparasites *Senotainia conica* were observed entering the burrows. Both females and males of *L. p. armatus* were detected on the flowers of *Achillea millefolium*, *Peucedanum oreoselinum*, *Daucus carota*, and *Tanacetum vulgare*. The article also includes phylogenetic relationships of Western Palearctic *Lindenius* species.

## Introduction

The genus *Lindenius* Lepeletier de Saint-Fargeau and Brullé, 1835 (Hymenoptera: Crabronidae^1^), contains 64 species, with most of them living in the Palearctic and a few in the Nearctic^2^. Representatives of this genus are medium-sized digger wasps similar to *Crossocerus* Lepeletier de Saint-Fargeau and Brullé, 1834, but differing from the latter by widely spaced hind ocelli and simple (edentate) mandibular apex. In Poland, the genus *Lindenius* is represented by four species^3^, the most common of which is *L. albilabris* (Fabricius, 1793). *L. pygmaeus* (Rossi, 1794) occurs in two subspecies: *L. p. pygmaeus* (Rossi, 1794) inhabiting northern Africa, the Iberian Peninsula, France and Italy, and *L. p. armatus* (Vander Linden, 1829) whose distribution covers the rest of Europe. The prey preferences of Nearctic species are basically unknown^4^, while the variation of prey within the best-studied Palaearctic species (*L. abilabris*, *L. panzeri* (Van der Linden, 1829), and *L. pygmaeus*) is very interesting^4^. Speaking specifically about *L. pygmaeus*, information on its nesting biology and food preferences is contained in the following papers: Marchal^5^ first listed the chalcid as a prey of *L. pygmaeus* and added a drawing of its larva and a cocoon; Ferton^6^ declared the subfamily Ophioninae (Hymenoptera: Ichneumonidae) as a prey, and Adlerz^7^ added Braconidae (Hymenoptera); Grandi^8^ described the structure of the nest, gave the dimensions of a single cell as 7 × 4 mm and listed the hymenopteran (Chalcididae) and dipteran prey; Maneval^9^ specified Ceratopogonidae, Sciaridae (Diptera) and Chalcididae, Braconidae and Formicidae (Hymenoptera) as a prey of *L. pygmaeus*. Later Minkiewicz^10^ described the nest architecture and replenish the list of prey by mostly Pteromalidae, but also Eulophidae and Torymidae (Hymenoptera). The number of prey items per cell ranged from 17^10^ to 42^8,11^. The egg of *L. pygmaeus*, having shape of an elongated crescent, is large in relation to the dimensions of the prey, and it is deposited at the base of the head near one of its limbs^10^. A detailed account of male behavior and intraspecific interactions in aggregations of *Lindenius* was presented by Miller and Kurczewski^12^. A few years later, same authors published observations of the nesting behavior of Nearctic species *L. armaticeps* (Fox, 1895), *L. buccadentis* Mickel, 1916 and *L. columbianus errans* (Fox, 1895), as well as a review of the world literature on the ethology of the Palearctic *L. albilabris*, *L. panzeri*, and *L. pygmaeus*^4^. The larva of *L. pygmaeus* was described by Grandi^8,11^, while *L. albilabris*—by Olszewski et al.^13^. An overview of nesting habits was presented by Kazenas^14^.

The genus *Lindenius* is also a good example of a group that poses taxonomic identification challenges. The problem with identifying species using the available keys—Kohl^15^, Beaumont^16^, Leclercq^17^ and the most recent study by Bitsch and Leclercq^18^ containing only 12 species—represents a real necessity to add to the knowledge of the taxonomy of this genus in its various aspects. None of the above-mentioned works allows the identification of all species of this genus. And yet any study of species behavior, ecology or phylogeny must begin with species identification. ^19^

Taxonomic identification of species is an important issue in biological research, because uncertain taxonomy of a species hinders research on its biology. Limitations in the description and identification of new species characterize a “taxonomic obstacle” that may be overcome using DNA-based methods, such as molecular barcoding^20,21^, phylogeny reconstruction^22,23^ and species delimitation^24–26^.

The purpose of this study is to supplement the existing information on the nesting biology of *L. p. armatus*, including (1) nesting behavior of females, (2) activity of females when bringing prey, (3) nest structure, (4) prey range, (5) accompanying parasitoids and kleptoparasites, (6) DNA barcode based identification of the collected material at the study site, and (7) preliminary reconstruction assessment of taxonomic issues related to a possible presence of cryptic species among the Western Palearctic *Lindenius* species, using the reconstruction of phylogeny and species delimitation, based on the DNA barcode data available to the authors.

## Results

### Observations of behavior

The nests of *Lindenius pygmaeus armatus* were found at two sites. The first one was the area of an esker and a sandy road in Sierakowo. The vegetation in the vicinity of this site was dominated by *Achillea millefolium* L.*, Artemisia vulgaris* L., *Berteroa incana* (L.) DC., *Cerastium holosteoides* Fr., *Daucus carota* L., *Geranium pusillum* L., *Lactuca serriola* L., *Peucedanum oreoselinum*L., *Potentilla anserina* L., *Tanacetum vulgare* L., *Taraxacum officinale* F.H. Wigg., and *Trifolium arvense* L. The nests were located close to each other (2 nests per 20 cm^2^) in non-vegetated areas. The second site with nests was a wasteland in Kowalewo Pomorskie among numerous nesting bees *Halictus tumulorum* (Linnaeus, 1758). The most dominant plants near the nests were the majority same plant species except for *Peucedanum oreoselinum* L.

**Figure 1:**
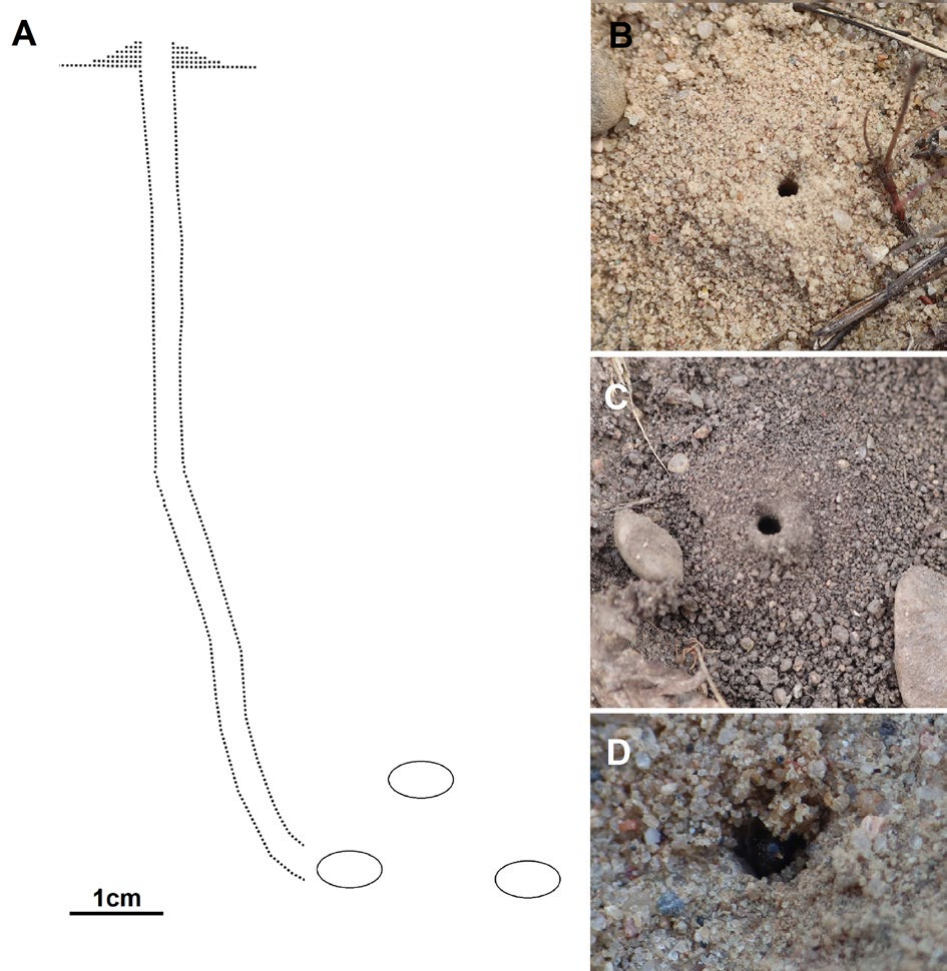
Nest of *Lindenius pygmaeus armatus*. (**A**) Lateral view of the nest and brood cells; (**B–D**) Top view of nest entrances.

**Figure 2:**
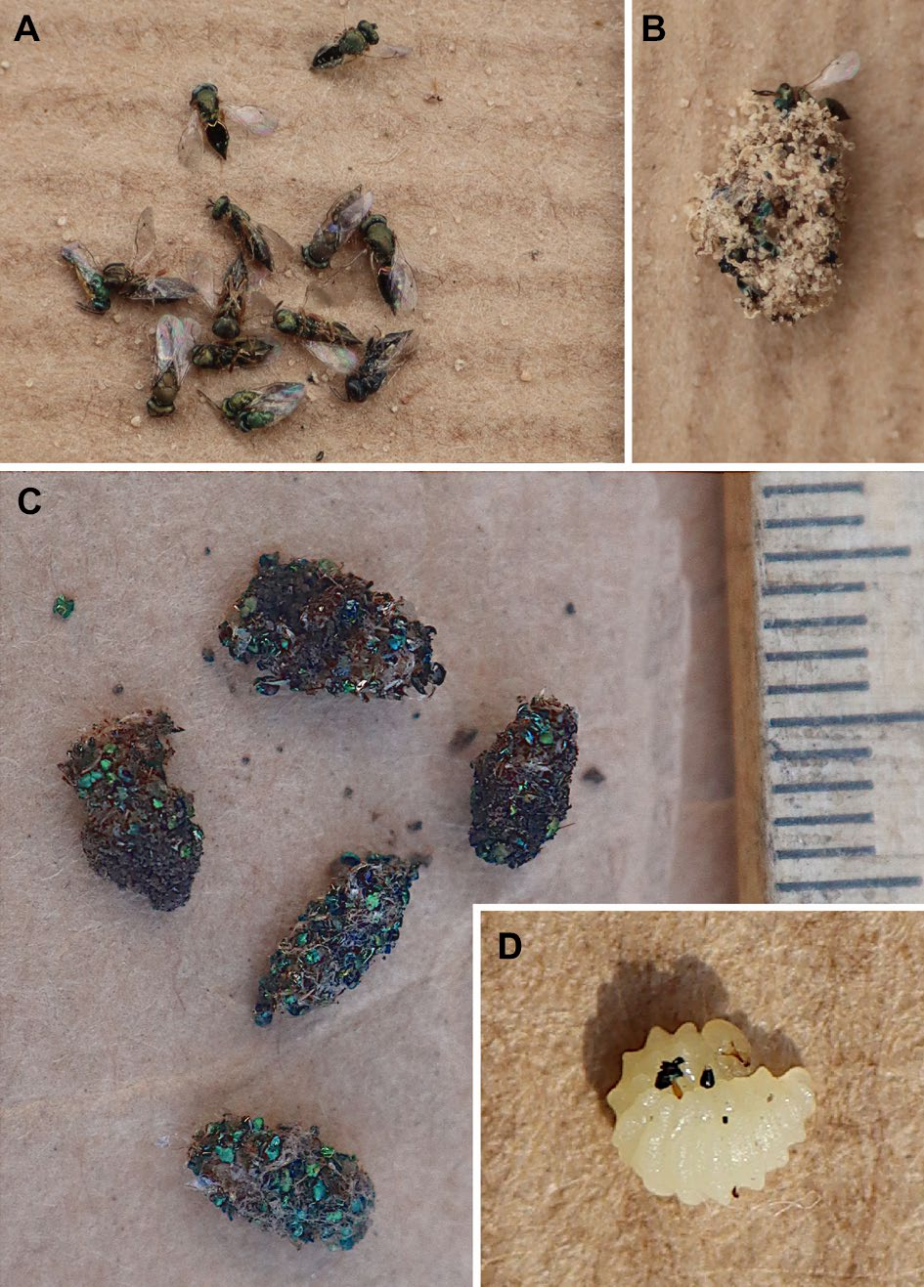
*Lindenius pygmaeus armatus*. (**A**) prey; (**B,C**) Cocoons; (**D**) Mature larvae.

**Figure 3:**
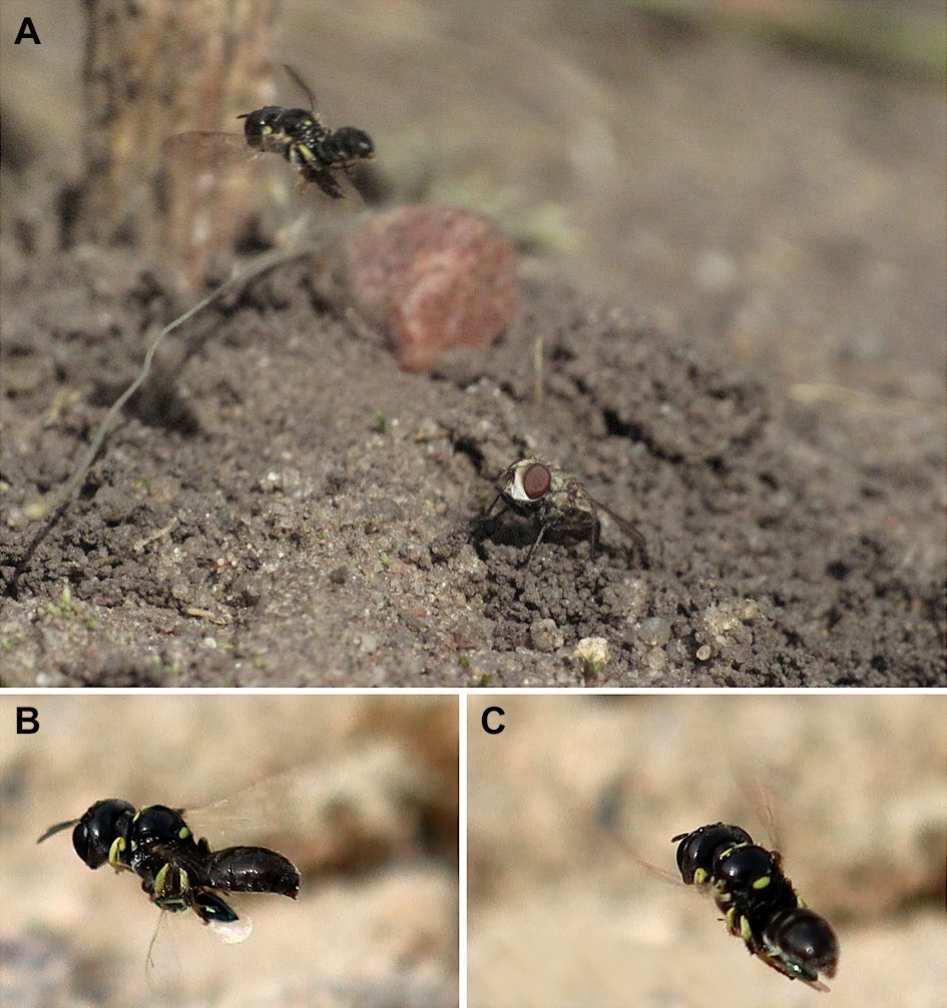
Adult of *Lindenius pygmaeus armatus*. (**A**) Female with prey at the nest entrance and *Senotainia conica*; (**B,C**) Female with prey in flight.

No copulation was observed. The process of digging the nest by the female lasted about 55 min (Fig. 1D, Sierakowo site). The tunnel of the burrow following the entrance was almost vertical to a depth of 5 cm, having then slightly diagonal position (at an angle of 10° to 20°, with a diameter of 2.5 mm); its total depth was about 10 cm. The cocoons were approximately 5–7 mm long and 2.5–3.5 mm wide. The burrow ended with a single cell, with other of them located in close proximity (Fig. 1A). The first nest contained 3 cells: one cell was provisioned with 14 prey items from the family of Pteromalidae, another one possessed a cocoon with an adult larva of *L. p. armatus* (a cocoon consisted of silk threads covered with fragments of chalcid wasps; Fig. 2B,C) and the third one was found with a pupa of a kleptoparasitic fly *Senotainia conica* (Fallén, 1810) (Diptera: Sarcophagidae). The second nest contained five cells with cocoons containing adult larvae of *L. p. armatus* (Fig. 2D). The excavated soil (while digging) formed a small mound around the nest entrance (Fig. 1B,C). The entrance to the nest was open during the entire provisioning period. No hunting females were observed, but they supported their prey in flight with their middle and hind legs (Fig. 3B,C). The females were extremely careful when entering the nest, even if there were no kleptoparasites inside: they approached the nest only after making many sudden and very winding detours, after which they had dived directly inside. The time of observation of females bringing prey to the nest was from 9.30 to 18.30. The frequency of provisioning the nest varied from 3 min to 15 min (usually about 5 min). When it was cloudy, the female waited out unfavorable conditions inside the nest. The last observation of the female with prey was on 8 August 2022. All prey items appeared to belong to nine species of Pteromalidae (Fig. 2A, Table 1). Based on field observations, the larva eats the provisions in about 5 days.

**Table 1:** Prey (Pteromalidae) of *Lindenius pygmaeus armatus* from the four observed nests.

Family	Species	Number of specimens		Share of all prey [in %]
		Female	Male	
Pteromalidae	*Coelopisthia extenta* (Walker, 1835)	2		6.06
	*Cyclogastrella simplex* (Walker, 1834)	2		6.06
	*Cyrtogaster vulgaris* Walker, 1833	4		12.12
	*Dibrachys cavus* (Walker, 1836)	1		3.03
	*Meraporus graminicola* Walker, 1834	5		15.15
	*Psychophagus omnivorus* (Walker, 1835)	5		15.15
	*Pteromalus* sp.	3	1	12.12
	*Stenomalina gracilis* (Walker, 1834)	7		21.21
	*Trichomalus perfectus* (Walker, 1835)	3		9.09
Total		32	1	100
		33		

During the absence of the female, the parasitoid *Myrmosa atra* Panzer, 1801 (Hymenoptera: Myrmosidae) was detected twice, and after the arrival of the female with its prey, the kleptoparasitic fly *Senotainia conica* entered the nest (Fig. 3A).

Adult wasps feed on flowers of the following plant species: *Achillea millefolium*, *Peucedanum oreoselinum*, *Daucus carota*, and *Tanacetum vulgare*.

### Phylogenetic relationships of Western Palearctic species

**Figure 4:**
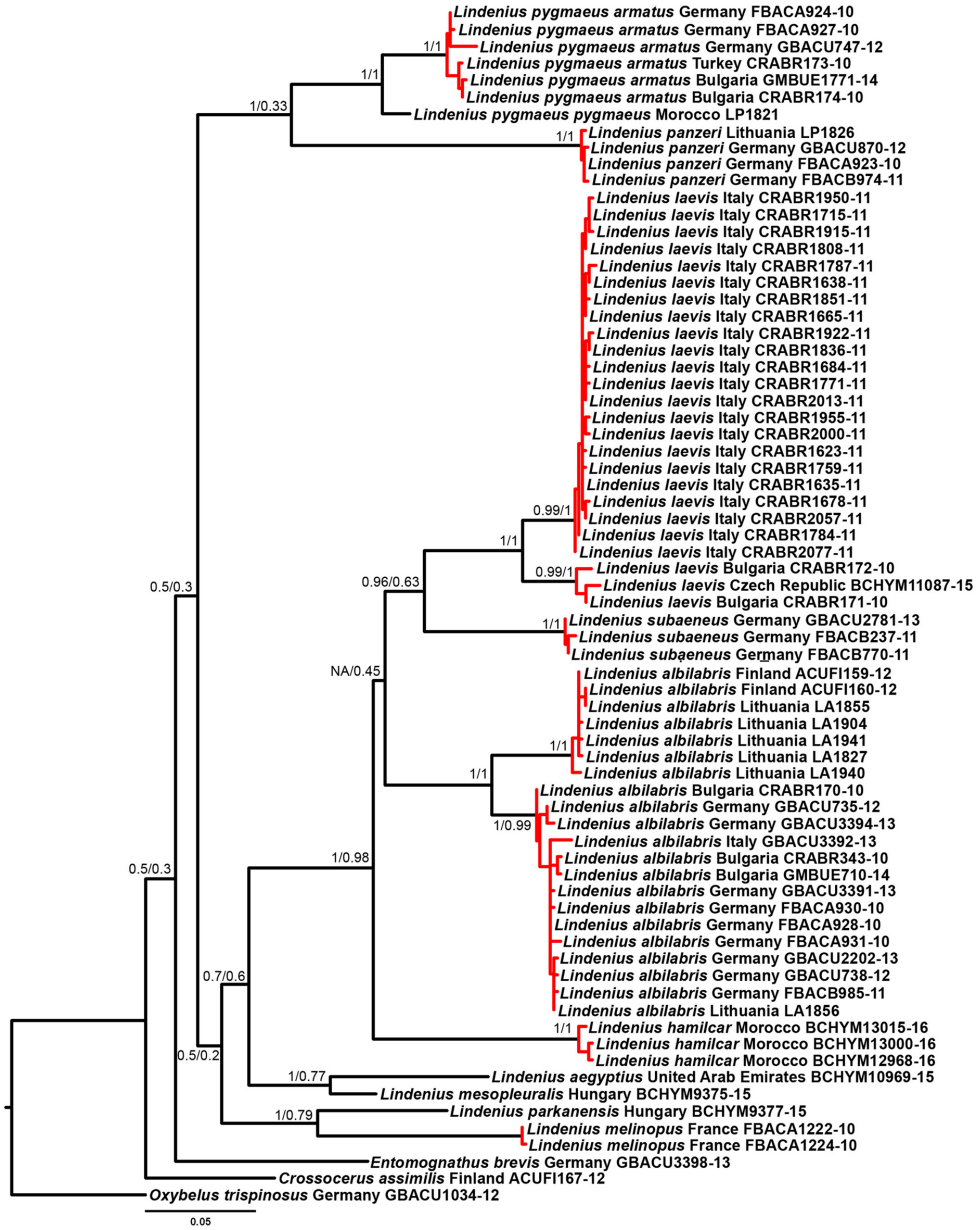
Phylogenetic relationships of Western Palearctic *Lindenius* COI mitotypes, reconstructed by the GTR + G + I evolution model and using the Maximum likelihood algorithm. Statistics of branches: Bayesian posterior probability, 1.000.000 generations/Maximum likelihood probability, 10.000 bootstrap replicates. Red clusters of the tree were supported as separate species by GMYC, bPTP, and ASAP delimitation algorithms. *Oxybelus trispinosus*, *Crossocerus assimilis*, and *Entomognathus brevis* were used as an outgroup.

A preliminary assessment of phylogenetic relationships of the studied wasp species, using the COI barcode sequences available to us, demonstrated that *Lindenius pygmaeus* is a sister clade of *L. panzeri*, a species provisioning the brood cells with Diptera. On the other hand, topology needs a taxonomic revision. Three of ten Western Palearctic representatives of the genus (*L. albilabris*, *L. laevis* A. Costa, 1867, and *L. pygmaeus*) were not delimited correctly as species by the delimitation algorithms GMYC, bPTP and ASAP (Fig. 4). Thus, our analysis demonstrated an ongoing speciation and possible recent divergence of cryptic species in this genus.

## Discussion

Fossil records of Crabronini are scarce and are known only from the Tertiary, extending from the Middle Eocene (Baltic amber species) to the Miocene (Dominican amber)^27^.

Significant progress has been made over the past decades in clarifying the phylogenetic relationships of the main Apoidea lineages^1,28^. The results of the research conducted by Sann et al.^1^ confirm the monophyly of each of the species-rich subfamilies: Astatinae, Bembicinae and Heterogynaidae, Crabroninae and Dinetinae, Philanthinae and the family Sphecidae. Phylogenetic analysis of the genus *Lindenius* has not yet been undertaken.

Regarding *Lindenius* in particular, the extant species are restricted to the Holarctic region, and one of them, *L. montezuma* (Cameron, 1891), reaches the Neotropical parts of southern Mexico^29^. Out of the three species of *Lindenius* found in Poland, *L. albilabris* preys almost exclusively on bugs (Hemiptera), *L. panzeri* mainly on Chloropidae flies (Diptera), and *L. p. armatus* on braconids and other Hymenoptera^10^. Ethological features can be a valuable supplement to the morphological characteristics of the digger wasp family^30^.

*L. p. armatus* nests are mainly found in sandy, sandy-clay, and loess (flat or slightly sloping) areas^8–11^, but they were also detected on soda ash postindustrial dumping grounds^31^, under high power lines^32^, and at a former airport^33^. Our results confirm the plasticity in the choice of nesting substrate as nests were observed both on heavily hardened soil (wastelands) and in sandy areas. During the study of the structure of *L. panzeri* nest, Abrahamsen^34^ found that the length and angle of inclination of the main tubule depended on the hardness of the substrate. Nests in all studied species are rarely built singly, usually a few at a time or in small colonies^13,35^. The species studied so far build nests at a depth of 3–12 cm in sand, fine gravel, loess or chalk^4^. At least 95% of the prey of *L. pygmaeus armatus* were Chalcidoidea and Ichneumonoidea (Hymenoptera), although females occasionally hunted small flies of Ceratopogonidae and Sciaridae (Diptera)^4^. The family most often hunted by *L. pygmaeus armatus* is Pteromalidae, followed by Eulophidae (Hymenoptera)^4^. The homogeneity of the food base in the conducted research (all belonging to Pteromalidae) illustrates this relationship well: for the first time, we detected two genera (*Cyrtogaster* Walker, 1833; *Psychophagus* Mayr, 1904) and five species: *Cyclogastrella simplex* (Walker, 1834); *Cyrtogaster vulgaris* Walker, 1833; *Psychophagus omnivorus* (Walker, 1835); *Stenomalina gracilis* (Walker, 1834), and *Trichomalus perfectus* (Walker, 1835) as the prey of *L. pygmaeus*; the four other taxa have been already known^4,10^.

It is also worth noting that almost all prey specimens were represented by females. Minkiewicz^10^, and Grandi^8^ also found that the majority of chalcidoids were females. Also, the pteromalid species hunted by *L. pygmaeus* are common taxa^36^. The evolution of nest seeking by *Lindenius* females has been attributed to factors such as the ability to form dense clusters, the tendency of related wasp species to initiate nests in pre-existing cavities, and a preference for nesting in heavily compacted soil. The search for nests of the same species by males and females may be an effective response to the pressure of kleptoparasitic flies^12^.

The behavior of both males and females can lead to intraspecific interactions. Interactions between females of the Nearctic species *L. columbianus* (Kohl, 1892) include entrance fights, attempted nest appropriation, prolonged nest visits, and temporary cooperation^12^. In the case of Palearctic species, Minkiewicz^10^ repeatedly observed a female of *L. panzeri* placing her prey in an adjacent nest if the entrance to her own nest was blocked.

In addition, the males of *L. panzeri* were observed waiting for females at the nest to perform copulation^37^. Unfortunately, no similar behavior was observed in *L. p. armatus* in this study.

The intraspecific interactions are certainly not fully understood and require thorough research, especially in the case of species nesting in clusters. Interactions in assemblages of this species may be an example of the initial stages of social evolution, developing through the mating of individuals of one generation^12^. Due to the very wide range of preferred prey (Hymenoptera and Diptera) of *L. p. armatus* and its nesting habitats, the status and the variation of this subspecies needs further focused exploration. In particular, a molecular study of specimens collected from different habitats in different parts of Europe is recommended.

Our preliminary species delimitation exercise supports the hypothesis that *L. p. armatus* may be considered an independent species. Although this hypothesis must be tested using a larger material and additional DNA markers, it is clear that the data on ecology and reproductive biology of *L. p. pygmaeus* and *L. p. armatus* must be analyzed separately.

Considering the probability of ongoing speciation among European *Lindenius* species, revealed by our phylogenetic analysis, the published data on wide ranges of prey, such as both Hemiptera and Diptera in *L. albilabris*, or Hymenoptera and Diptera in *L. pygmaeus*, must be critically reanalyzed. It is possible that the available data must be re-assorted among the undescribed yet cryptic species, which may actually have more specialised niches and a narrower prey choice than previously thought. Our study demonstrates that even in the central Europe with a comparatively very well studied fauna the ethological and ecological observations of insects must be accompanied by the molecular identification of species.

## Methods

### Field observations

Observations of nesting *Lindenius pygmaeus armatus* were carried out on the wasteland in Kowalewo Pomorskie (53°10'05.7” N; 18°52'15.5” E) and Sierakowo (53°10'18.9" N; 18°52'21.7" E). The research was conducted from early June to mid-August (2020–2022) on sunny and warm days with a temperature of at least 18 °C. The nests were examined by digging at the stage when the female stopped bringing prey to the nest. While the nest was being examined, its structure was drawn on graph paper. A kleptoparasitic fly pupa found in the cell was placed in an Eppendorf tube and kept until the imago emerged. The pteromalid prey found in the nests was placed in plastic tubes with 70% ethyl alcohol and then identified. The behavior of females, nest digging and provisioning of cells were analyzed using video recordings taken with a Canon CCD-V800E 10X camera and direct observations. Photographs were taken using additional Raynox M-250 macro lens.

### Phylogenetic reconstruction

For the assessment of phylogenetic relationships of the study object, we downloaded the mitochondrial DNA cytochrome c oxidase subunit 1 (mtDNA COI) barcodes of all Western Palearctic *Lindenius* mitotypes available from the Barcode of Life Data System (www.boldsystems.org) Barcode Index Number (BIN) database^20,21^. The sequences of *Oxybelus trispinosus* (Fabricius, 1787), *Crossocerus assimilis* (F. Smith, 1856), and *Entomognathus brevis* (Vander Linden, 1829) from the same database were used as an outgroup. The dataset was supplemented by a few other *Lindenius* barcodes, which were obtained according to the methodology of DNA extraction, polymerase chain reaction, and sequencing by Budrys et al.^38^. At the start of analysis, the nucleotide substitution model most likely for the obtained alignment was selected from the list of 24 models available in MEGA 11^22^. Phylogeny testing was proceeded using 10.000 replicates of the bootstrap procedure. Substitution patterns were best described by the general time reversible model with gamma distribution of evolutionary rates, using four discrete categories, and a fraction of invariable sites (GTR+G+I). This model was applied for reconstruction of phylogenetic relationships using the Bayesian inference by means of MrBayes 3.2.7a^23^, and using the Maximum likelihood algorithm, implemented in MEGA 11; for the Bayesian inference, Markov Chain Monte Carlo (MCMC) analyses were run for 1.000.000 generations. For species delimitation, three algorithms were applied: the single threshold General Mixed Yule Coalescent model (GMYC)^24^, implemented as “splits” package in R environment, the Bayesian Poisson Tree Processes method (bPTP) web server (species.h-its.org/ptp/)^25^, and the Assemble Species by Automatic Partitioning (ASAP) web server (bioinfo.mnhn.fr/abi/public/asap/asapweb.html)^26^. The ultrametric dichotomous trees for the GMYC analysis were obtained using the RelTime-ML algorithm, implemented in MEGA 11. The nomenclature of the digger wasps follows^2^, chalcid wasps^39^, plants^40^.

## Data Availability

The datasets used and/or analysed during the current study are available from the corresponding author on reasonable request.
